# Supplementary material for: Enhanced glacial lake activity threatens numerous communities and infrastructure in the Third Pole
Source: Nat Commun. 2023 Dec 12;14:8250. doi: 10.1038/s41467-023-44123-z (PMC10716169; doi:10.1038/s41467-023-44123-z)
Supplement: Supplementary file 1 — Supplementary Information [file 41467_2023_44123_MOESM1_ESM.pdf]

Supplementary Materials

Enhanced glacial lake activity threatens numerous communities and infrastructure in the Third Pole

Taigang Zhang [1,2,3], Weicai Wang [1*], Baosheng An [1,4], Lele Wei [1,2,3],

[1] State Key Laboratory of Tibetan Plateau Earth System, Environment and Resources (TPESER), Institute of Tibetan Plateau Research, Chinese Academy of Sciences, Beijing 100101, China

[2] College of Earth and Environmental Sciences, Lanzhou University, Lanzhou 730000, China

[3] Center for the Pan-Third Pole Environment, Lanzhou University, Lanzhou 730000, China

[4] School of Science, Tibet University, Lhasa 850011, China

✉ e-mail: weicaiwang@itpcas.ac.cn

This file includes:

Supplementary Figures 1 to 10
Supplementary Tables 1 to 7
Supplementary References

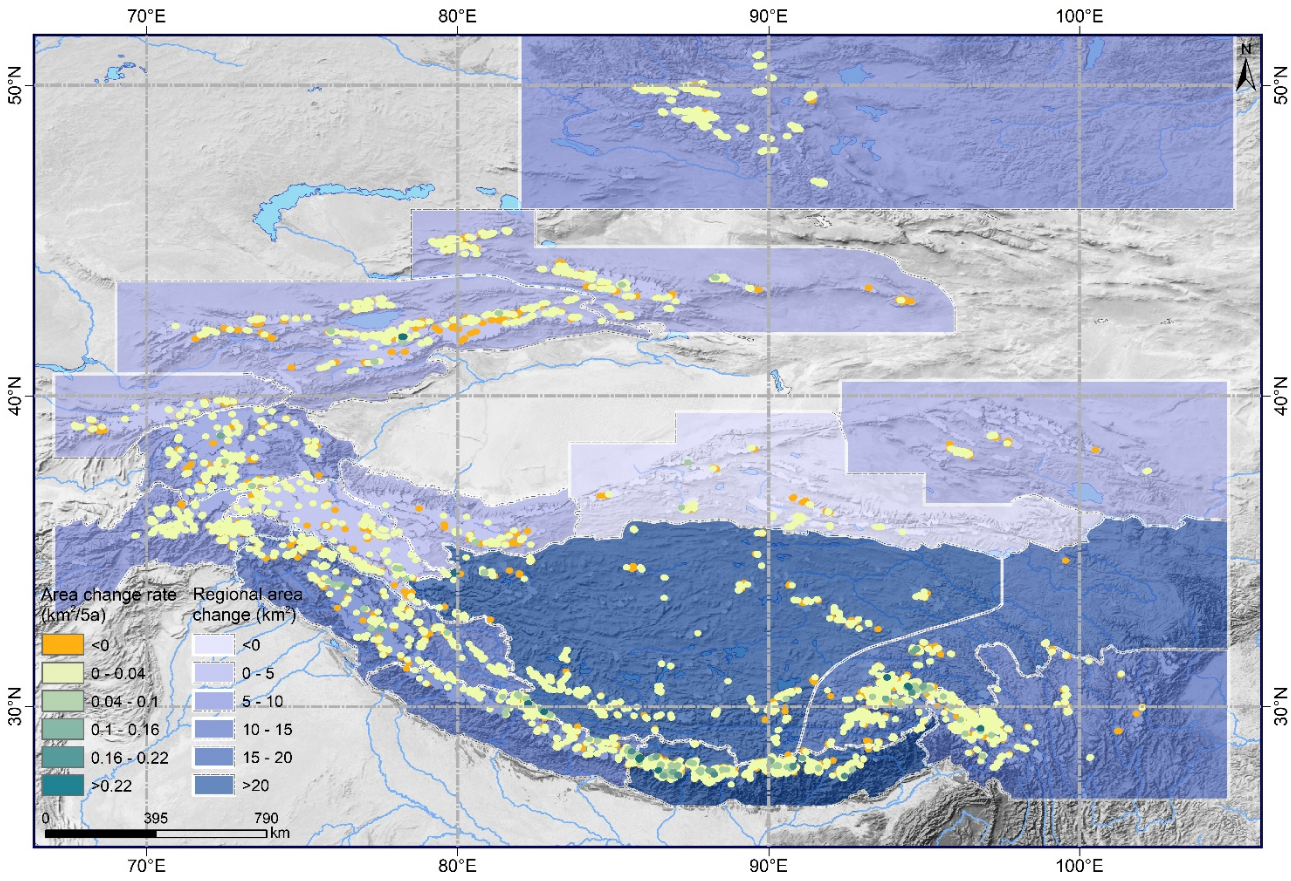

Supplementary Figure 1 | Glacial lake distribution and changes in the Third Pole between 1990 and 2018. These long-term trends were derived from the glacial lake dataset published by Wang et al. (2020) in order to compare them with our short-term trends during 2018–2022. This figure corresponds to the figure1 in the main article.

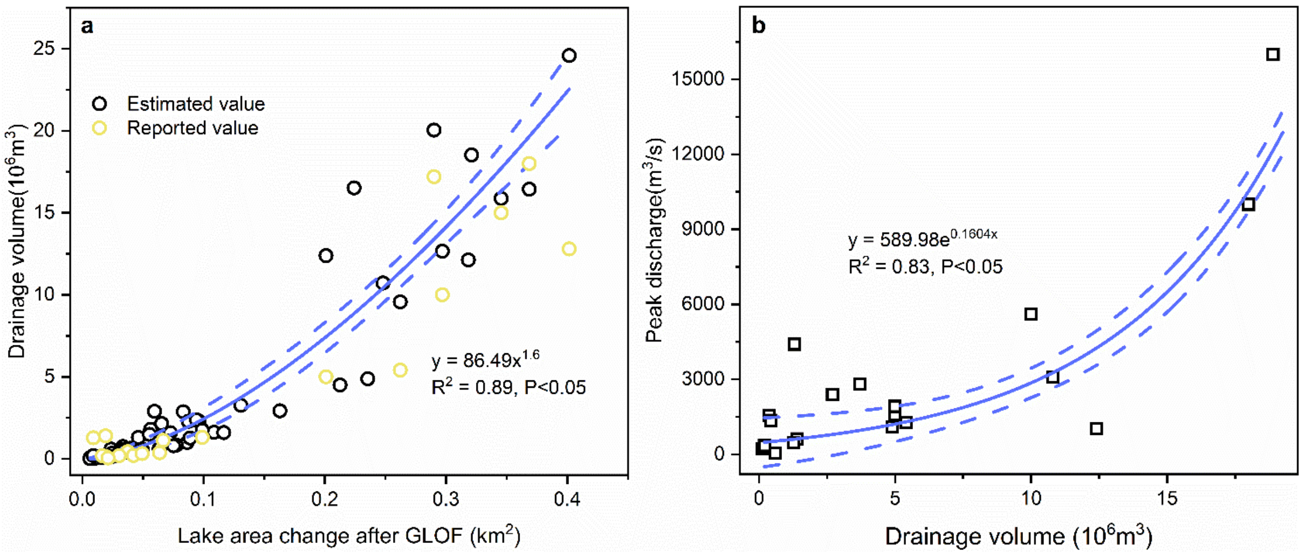

Supplementary Figure 2 | Drainage volume and peak discharge of GLOFs. a We reconstructed the drainage volume of the historical GLOFs that occurred after 1980 based on the glacial lake area change before and after the outburst and the lake volume-area relationship.

The estimated values were verified using reported real values. **b** The relationship between the peak discharge and drainage volume of the GLOFs is also shown, which is important for GLOF modeling. The basic data used for fitting these curves are presented in Supplementary Tables 2, 3, and 7.

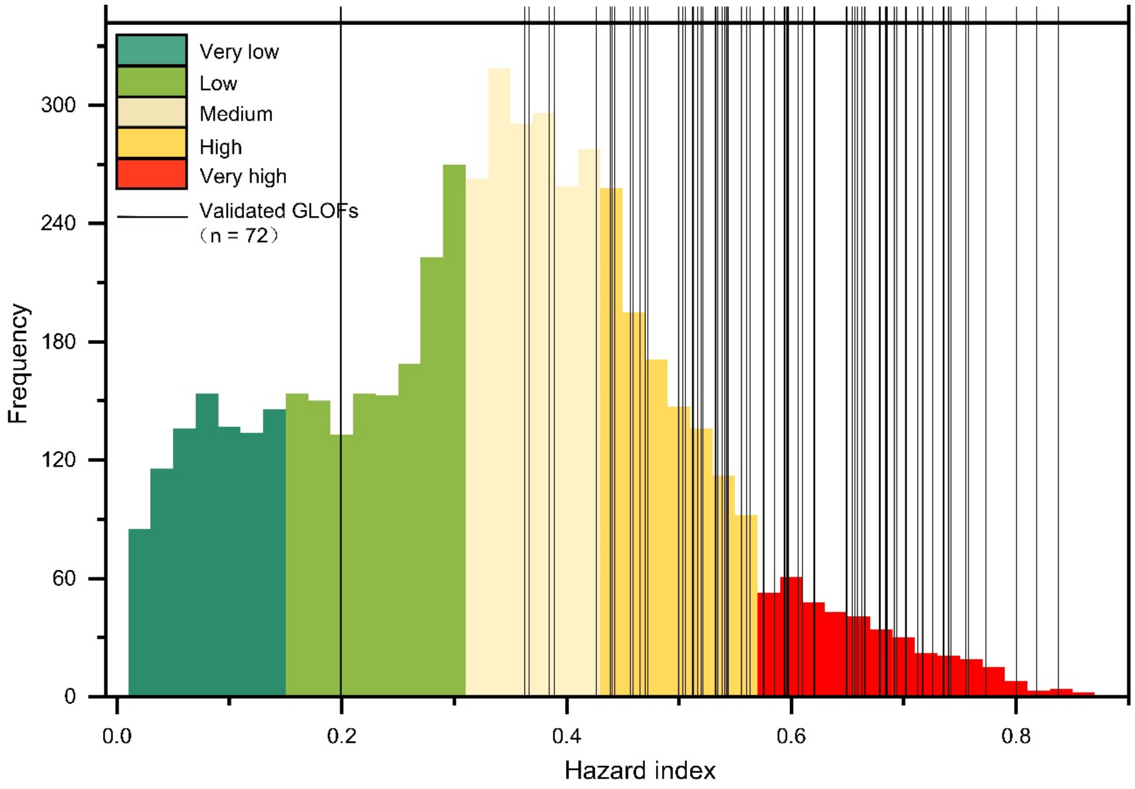

Supplementary Figure 3 | Hazard index of glacial lakes in the Third Pole. The histogram presents the classifications of the hazard levels of the glacial lakes, which were determined using the natural Jenks classification method in ArcGIS. Thin vertical lines denote the hazard index of 72 validated GLOFs.

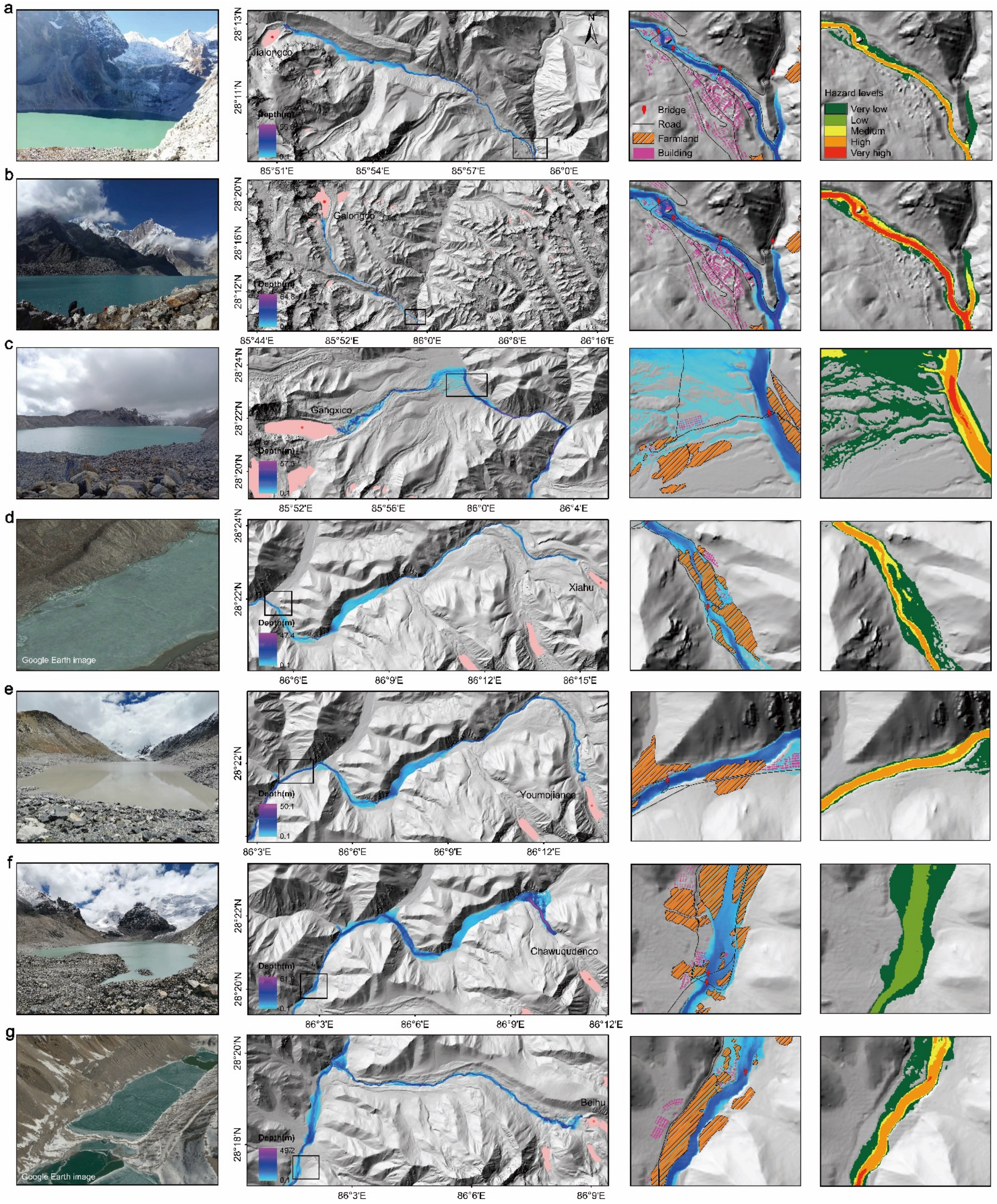

Supplementary Figure 4 | Seven glacial lakes that pose a very high risk in the Poiqu River Basin in the China-Nepal border area were selected to exhibit the simulated GLOFs, downstream exposure, and mapped hazards: (**a**) Jialongco, (**b**) Galongco, (**c**) Gangxico, (**d**) Xiahu, (**e**) Youmojanco, (**f**) Chawuqudenco, and (**g**) Beihu. The photos of Youmojanco and Chawuqudenco were provided by Miaomiao Qi. The screenshots of Xiahu and Beihu taken from Google Earth.

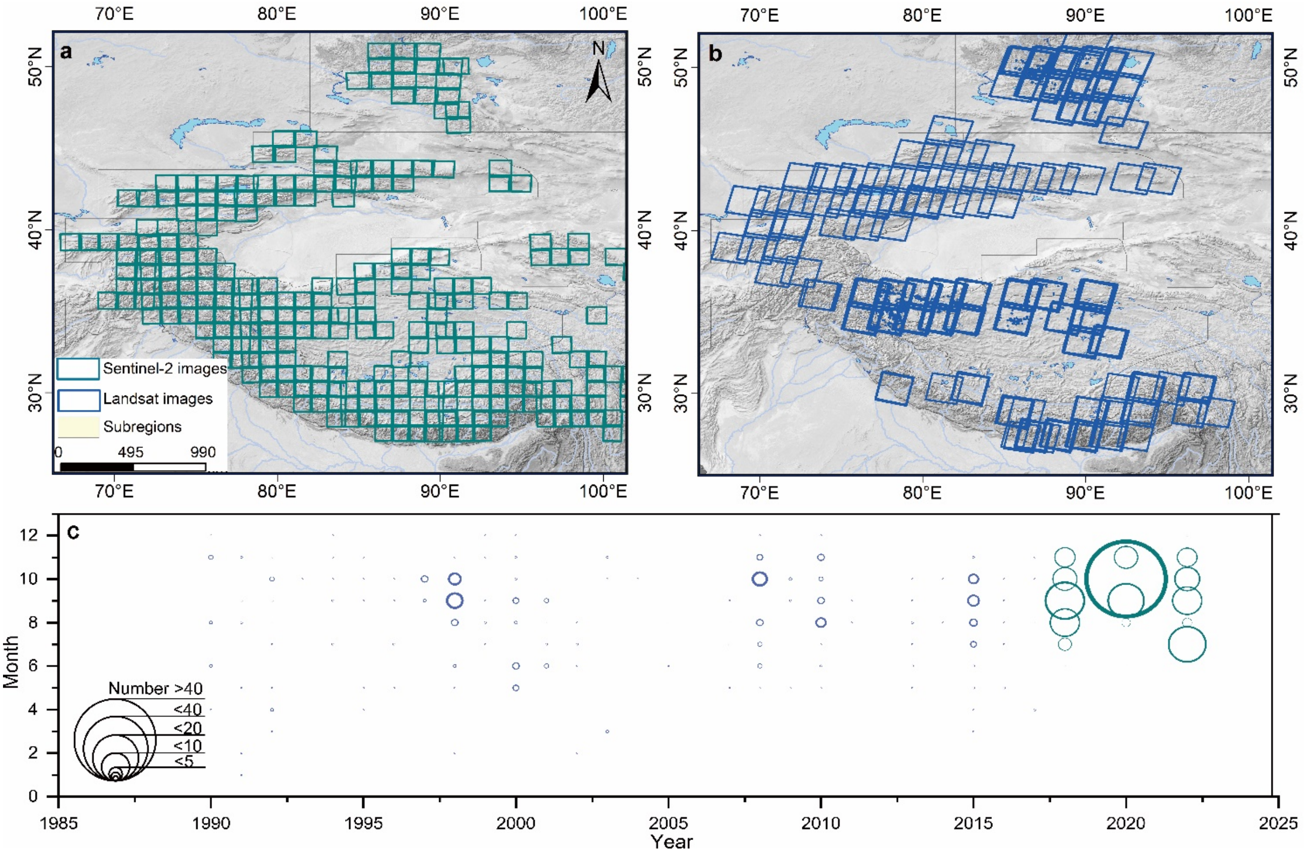

Supplementary Figure 5 | Overview of Landsat and Sentinel images used for mapping the glacial lakes. a A total of 878 Sentinel-2A/B images covering all of the glaciated regions in the Third Pole were used to delineate the glacial lakes in 2018, 2020, and 2022. **b** 313 individual scenes from Landsat missions 4, 5, 7, and 8 were used to identify glacial lakes overlooked in other available glacial lake datasets. **c** Temporal distribution of used remote sensing images.

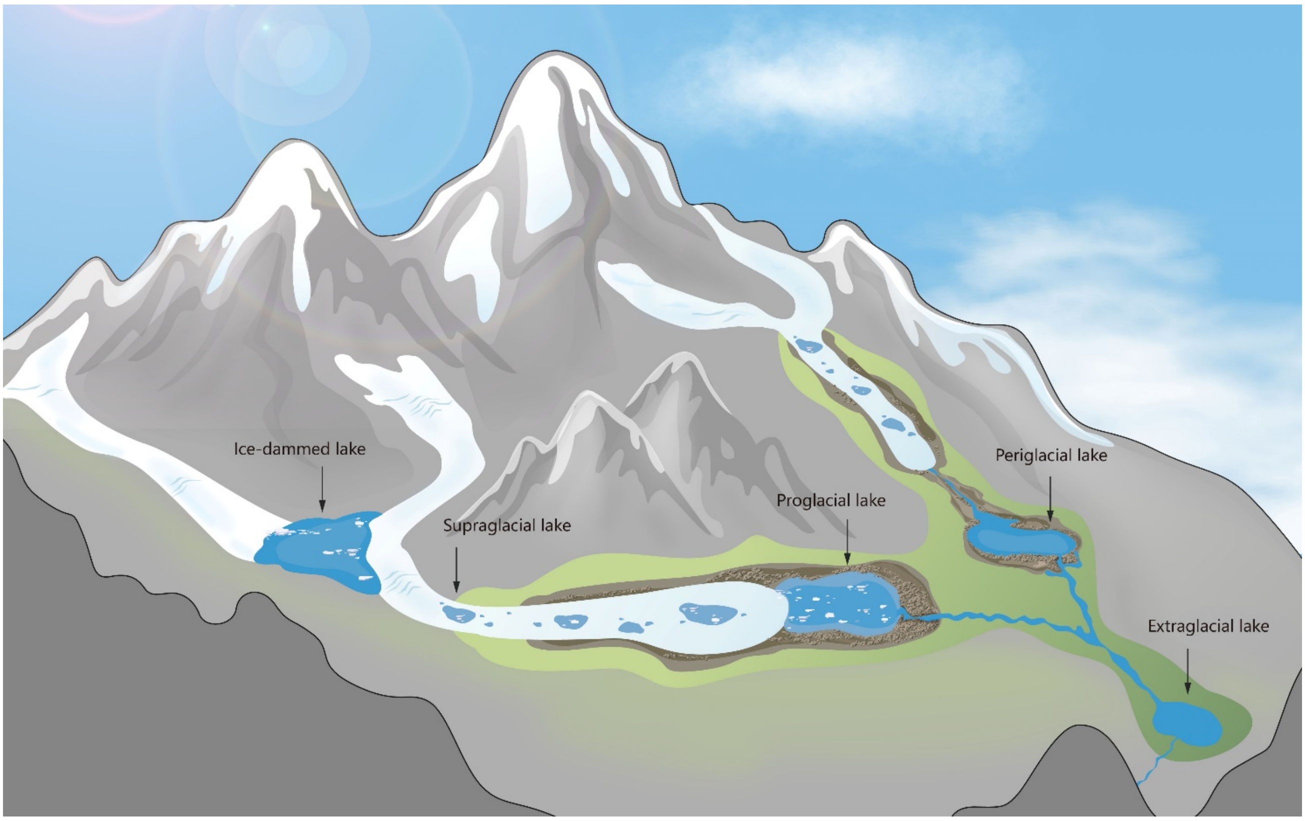

Supplementary Figure 6 | The various types of glacial lakes. Based on their topological positions relative to their parent glaciers, the glacial lakes are classified into five types, namely, proglacial, periglacial, extraglacial, supraglacial, and ice-dammed lakes.

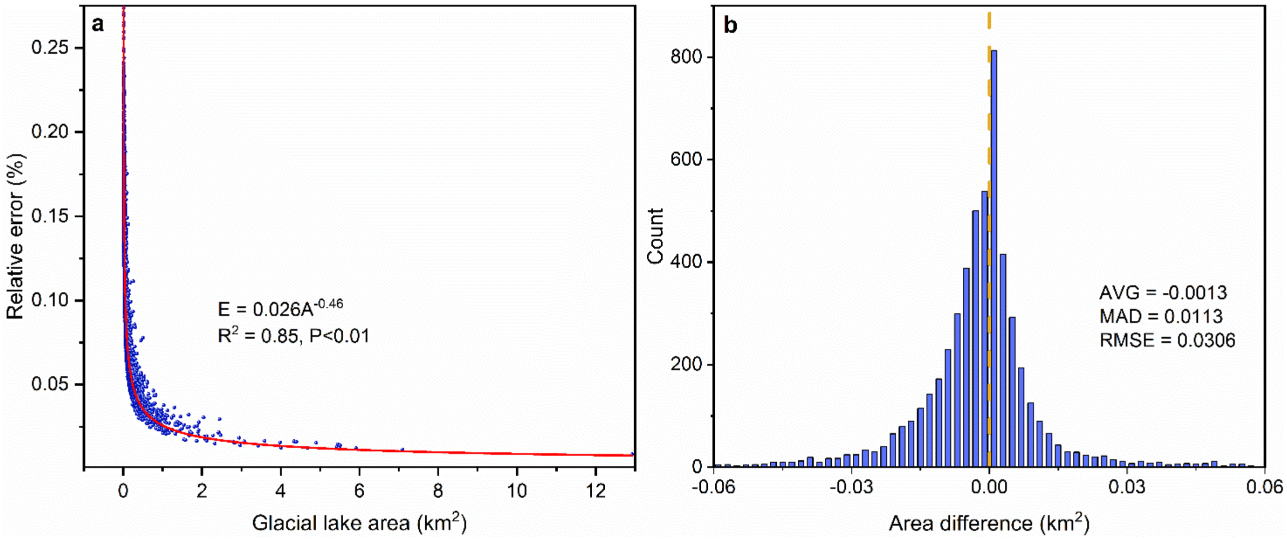

Supplementary Figure 7 | Uncertainty of glacial lake delineation. a The relationship between the relative area error and the size of the glacial lakes in the Third Pole is examined. **b** The glacial lakes mapped from Sentinel-2A/B images in 2018 are compared individually with the glacial lake dataset provided by Wang et al. (2018) for the same year. The average deviation (AVG), mean absolute deviation (MAD), and root mean square error (RMSE) were selected to depict these differences.

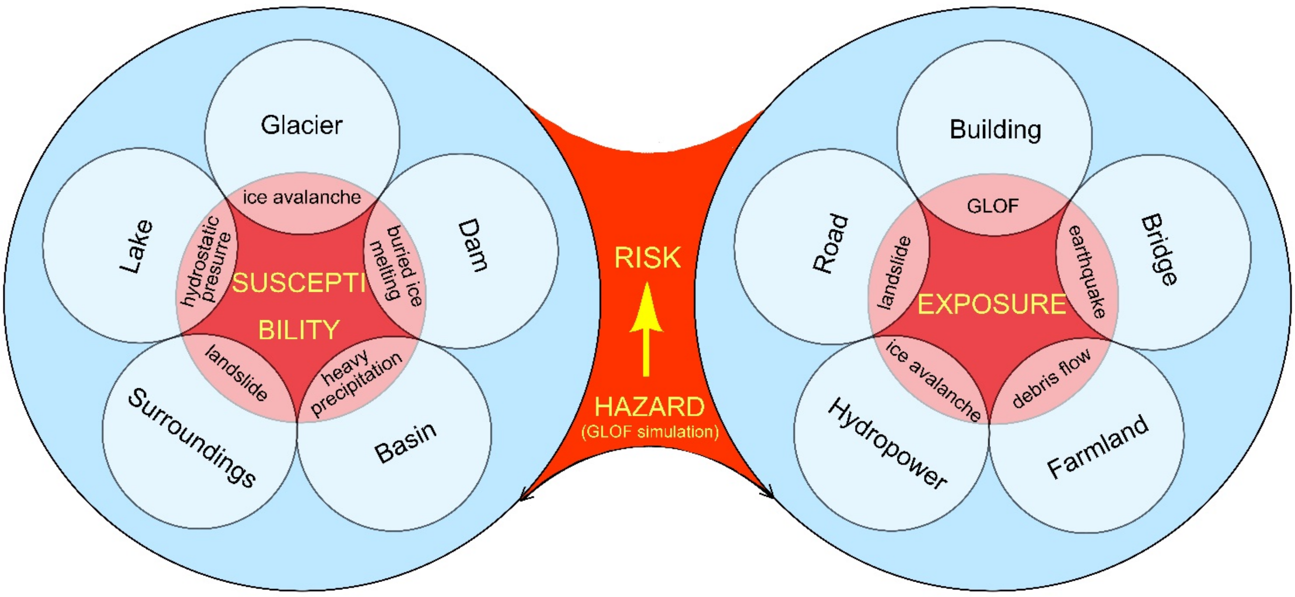

**Supplementary Figure 8 | ** Conceptual models for hazard, exposure, and risk assessment of glacial lakes.

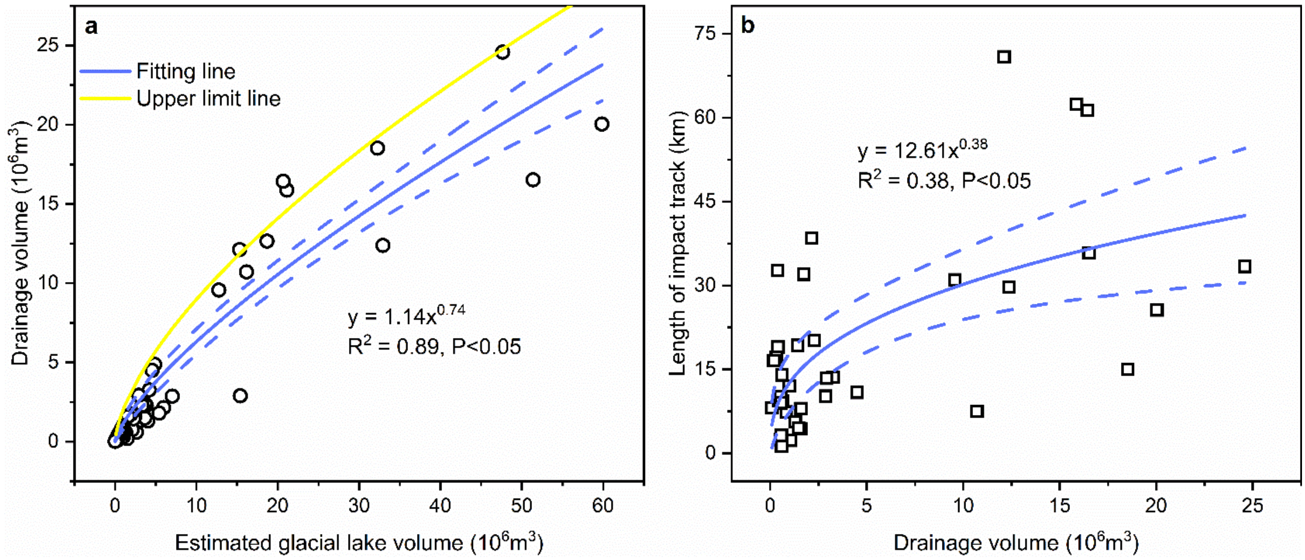

Supplementary Figure 9 | Estimates of drainage volume and length of impact track for GLOF simulation. a Relationship between drainage volume and the glacial lake volume. **b** Relationship between GLOF impact length and the drainage volume.

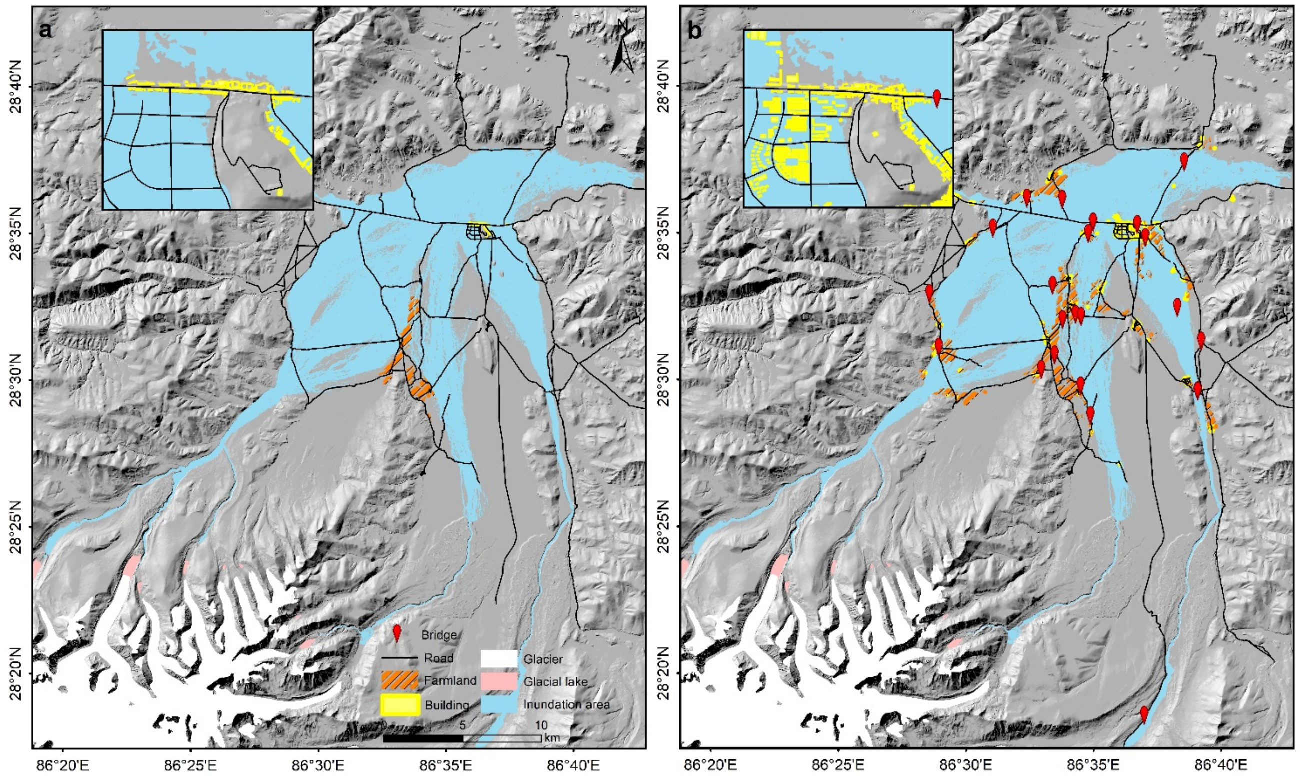

Supplementary Figure 10 | The Gangga town (28°35′N, 86°34′E) area on the north slope of Everest was chosen as an example to showcase the uncorrected (**a**) and corrected (**b**) downstream exposure information of the glacial lakes.

Supplementary Table 1 | The state of the glacial lakes with respect to their number, area, volume, and area change rate in the Third Pole in 2022. These features were aggregated into 16 Global Terrestrial Network for Glaciers (GTN-G) regions. The glacial lake area expansion rates reflets a mean level from 2018 to 2022.

Region	Number	Area (km^2)	Volume (km^3)	Area change rate (km^2/5a)
Altai	262	32.43 ± 1.97	0.45 ± 0.35	0.45 ± 0.17
East Tianshan	342	22.45 ± 1.86	0.29 ± 0.24	1.32 ± 0.45
West Tianshan	424	39.91 ± 2.47	0.96 ± 0.8	-1.65 ± 0.67
Hissar Alay	71	5.87 ± 0.41	0.08 ± 0.06	0.06 ± 0.06
Pamir	291	40.74 ± 2.1	0.66 ± 0.47	0.36 ± 0.38
Hindu Kush	292	29.95 ± 1.94	0.42 ± 0.33	-0.38 ± 0.33
Karakoram	253	18.6 ± 1.43	0.29 ± 0.24	-1.23 ± 1.14
West Kunlun	93	11.1 ± 0.65	0.34 ± 0.29	0.28 ± 0.14
Inner Tibet	714	93.84 ± 4.59	2.89 ± 2.35	2.03 ± 1.15
East Kunlun	111	9.58 ± 0.7	0.16 ± 0.13	-0.29 ± 0.21
Qilian Shan	49	6.03 ± 0.36	0.12 ± 0.11	0.18 ± 0.03
Western Himalaya	491	39.62 ± 2.93	0.69 ± 0.58	1.61 ± 0.46
Central Himalaya	325	49.31 ± 2.41	2.16 ± 1.95	1.64 ± 0.47
Eastern Himalaya	1,119	192.49 ± 9.42	6.72 ± 6	10.24 ± 1.74
Southeastern Tibet	479	81.47 ± 3.78	2.34 ± 1.97	4.78 ± 0.85
Henduan Shan	578	75.39 ± 4.14	1.53 ± 1.25	3.27 ± 0.59
Total	5,894	748.79 ± 41.16	20.13 ± 17.12	22.68 ± 8.84

Supplementary Table 2 | An inventory of glacial lake outburst floods (GLOFs) originating from moraine-dammed lakes in the Third Pole since 1900. These events are primarily selected from available GLOF datasets. Two GLOFs are reported first in this study. To ensure the credibility of the GLOFs, we examined in detail the documentary sources of each event, as well as verifying their remaining topographical evidence through Google Earth images, such as breached dam, pre-GLOF water levels, and outwash fans. Dozens of events were excluded because of insufficient geomorphic features or not produced by moraine-dammed lakes. For the GLOFs with unreliable date were not collected.

ID	Country	Lake name	Outburst date	Longitude (°)	Latitude (°)	Triggers	Reported drainage volume ($10^6 m^3$)	Reported peak discharge ($m^3 s^{-1}$)	References
1	China	Taraco	1938/8/28	86.1311	28.2956	Buried ice melting	6.3		1
2	China	Qiongbihema Tsho	1940/7/10	88.9211	27.8469	Ice avalanche	12.4	1036	1
3	China	Sangwangco	1954/7/16	90.1031	28.2308	Ice avalanche	300	10000	1,2
4	China	Lureco	1950s	90.5892	28.2731				3
5	China	Cirenmaco	1964	86.0656	28.0669				4,5
6	China	Cirenmaco	1981/7/11	86.0656	28.0669	Ice avalanche		16000	1
7	China	Cirenmaco	1983	86.0656	28.0669				4,5
8	China	Longda Tsho	1964/8/25	85.3492	28.6169	Ice avalanche	10.8	3100	1
9	China	Gelhaipuco	1964/9/21	87.8097	27.9636	Ice avalanche	23.4		1
10	China	Damenlahaico	1964/9/26	93.0411	29.8675	Ice avalanche	3.7	2812	1;6
11	China	Ayaco	1968/8/15	86.4931	28.3472				4
12	China	Ayaco	1969/8/17	86.4931	28.3472				4
13	China	Ayaco	1970/8/18	86.4931	28.3472		90		1
14	China	Pogeco	1972/7/23	94.725	31.7353	Ice avalanche			1
15	China	Bogeco	1974/7/6	94.7597	31.8575	Ice avalanche			4
16	China	Zarico	1981/6/24	90.6069	28.2986	Ice avalanche			1
17	China	Yindapuco	1982/8/27	87.9083	27.9519	Ice avalanche	12.8		1,4
18	China	Ganxico	1988/7/15	96.5022	29.4631	Ice avalanche	5.4	1270	2,7
19	China	Xiaga	1995/5/26	91.9361	28.8022	Ice avalanche		38.3	8
20	China	Zanaco	1995/6/7	85.3703	28.6619	Ice avalanche			4
21	China	Chongbaxiaco	2000/8/6	89.7425	28.2119	Ice avalanche			4
22	China	Jialongco	2002/5/23	85.8483	28.2117	High temperature and/or heavy precipitation			4,9
23	China	Jialongco	2002/6/29	85.8483	28.2117	High temperature and/or heavy precipitation			4,9
24	China	Degaco	2002/9/18	90.6736	28.3331	Ice avalanche			4
25	China	Langco	2007/8/10	91.8061	27.8275	High temperature and/or heavy precipitation			10
26	China	Zhemaico	2009/7/3	92.3425	28.0144	High temperature and/or heavy precipitation			3
27	China	Coga	2009/7/29	94	30.8292	High temperature and/or heavy precipitation			4
28	China	Geiqu	2010	87.985	27.9506	High temperature and/or heavy precipitation			4
29	China	Gongbatongshaco	2016/7/5	86.0636	28.0778	Ice avalanche	1.4	618	11
30	China	Zangla Tsho	1994/4-1994/10	82.1167	30.3561	High temperature and/or heavy precipitation			12
31	China	Langbu Tsho	1992/9-1992/11	86.4206	27.9261	Ice avalanche			12
32	China	Ranzeranco	2013/7/5	93.5356	30.4764	Ice avalanche	15		13
33	China	Gebumaco	1991/6/12	96.5561	29.7525	Upper GLOF			2
34	China	Dalonggongba	2005/9/1	96.4608	29.7528	High temperature and/or heavy precipitation			2
35	China	Rejieco	1992/9-1992/10	88.8947	27.965	Ice avalanche			2
36	China	Zhalonggabu	1995-1996	85.4761	28.6617	Ice avalanche	1.12		2,14
37	China	Oubuguoco Upper	2003-2004	93.5506	29.63	Ice avalanche			2
38	China	Nalongzangbu Tributary	2014/6/1	94.9444	30.5317	Ice avalanche			2

39	China	Tulaco	2015/7/3	94.2531	30.7367	Buried ice melting			2
40	China	Jichudrake North 1	Before 1966	89.3125	27.8769	Ice avalanche			15
41	China	Jichudrake North 2	Before 1967	89.3508	27.8831	Ice avalanche			15
42	China	Upper Jiejiu Tsho	Before 1966	90.7122	28.2722	Ice avalanche			15
43	China	Upper Shegong Tsho	Before 1966	90.7403	28.3039	Ice avalanche			15
44	China	Cuoalong glacier lake	1955-1966	90.5764	28.0622	Ice avalanche			15
45	China	Sanga North	1987	93.8975	30.1269				14
46	China	Unnamed 2nd	1996-1997	92.3875	27.7047				14
47	China	Dareco Sourth	2001-2003	85.9189	28.1381	Ice avalanche			14
48	China	Jitang Sourth	2002-2003	94.3239	30.6831				14
49	China	Yindapuco upper	2015/10/1	87.8964	27.9431				14
50	China	Zanglaco East	2015/09-2016/09	82.2	30.31				14
51	China	Sangwangco East	2007/11-2008/10	90.23	28.28				14
52	China	Baitang Weat	1988/10-1992/10	92.7853	29.5528				14
53	China	Dacangco	1956/6/1	86.3508	28.1925	High temperature and/or heavy precipitation		2408	16
54	China	Zanglaco West	1994/12/30-1995/10/30	81.9989	30.3753				17
55	China	Jinwuco	2020/6/26	93.6317	30.3564	Landslide	10	5602	18
56	China	Pasu Lake	2008/1/1	74.8797	36.4575	Ice avalanche			19
57	China	Pasu Lake	2008/4/1	74.8797	36.4575	Ice avalanche			19
58	China	Unknow2	2018/06-2018/10	86.3211	28.2447				This study
59	China	Unknow1	2017/06-2017/10	98.0036	28.7169				This study
60	Myanmar	Hezhen South	2008/11-2009/10	97.8364	28.3208				14
61	Bhutan	Upper Chokham Tsho	Before 1966	89.3683	27.8356	Ice avalanche			15
62	Bhutan	Unnamed 1st	Before 1966	89.8944	28.0347	Ice avalanche			15
63	Bhutan	Simdong Goi Tsho	Before 1966	89.8114	28.2164				15
64	Bhutan	Jhomohari South	Before 1966	89.2672	27.7886	Ice avalanche			15
65	Bhutan	Tarikha Lake	Before 1956	90.6733	28.0281				5
66	Bhutan	Chubda Tsho	Before 1956	90.6969	28.0128	Ice avalanche			15
67	Bhutan	upper Gangri Tsho1	1998	90.8161	27.8961	Ice avalanche			15
68	Bhutan	upper Gangri Tsho2	1998	90.8161	27.8961	Upper GLOF			17
69	Bhutan	A lake in Jangothang	1966-1974	89.3461	27.82	Ice avalanche			15
70	Bhutan	Tarina Tsho	1957	89.8989	28.1044	Ice avalanche			15
71	Bhutan	Upper Luggye	1990-1991	90.3375	28.0892				14
72	Bhutan	Upper Luggye	2009-2010	90.3375	28.0892				14
73	Bhutan	Unamed 3rd	1997-1998	90.4286	27.9011	Landslide			14
74	Bhutan	Luggye Tsho	1994/10/7	90.2964	28.0925	Buried ice melting	17.2		20
75	Bhutan	Lemthang Tsho	2015/6/28	89.5806	28.0686	Landslide	0.37	1562	20
76	Nepal	Nare Lake	1977/9/3	86.8747	27.8369	Buried ice melting	4.9	1100	20,21
77	Nepal	Nagma Pokhari	1980/6/23	87.8639	27.8653	Landslide			22
78	Nepal	Dig Tsho	1985/8/4	86.5856	27.8744	Ice avalanche	5	1600	23
79	Nepal	Dig Tsho	2015/4/25	86.5856	27.8744	Ice avalanche			22
80	Nepal	Chubung	1991/7/12	86.4658	27.8856	Landslide			24
81	Nepal	Chukhung	Before 1962	86.8875	27.8886		5	1915	25
82	Nepal	Tam Pokhari	1998/9/3	86.8444	27.7414	Ice avalanche	18	10000	20,26

83	Nepal	Langmale lake	2017/4/20	87.1381	27.8111	Landslide	1.3	4400	22
84	Nepal	Zanglha North	1998-1999	86.7769	27.9511				14
85	Nepal	Lhotse Glaicer lake	2015/5/25	86.8983	27.9058	Buried ice melting			27
86	Nepal	Lhotse Glaicer lake	2016/6/12	86.8983	27.9058	Buried ice melting		210	27
87	Nepal	Tiptola	1963	87.7494	27.8153	Ice avalanche			28
88	Nepal	Tiptola	1968	87.7494	27.8153	Ice avalanche			28
89	Nepal	Unamed 4th	2010/06/09-2012/10/23	86.6683	27.9453				17
90	Nepal	Changri Shar Glacier lake	2017/7/15	86.8042	27.9742				29
91	India	Choradari	2013/6/7	79.0608	30.7472	High temperature and/or heavy precipitation	0.43	1352	30,31
92	India	Kongyangmi La Tsho	1997/4-1997/10	88.7811	27.9006	Ice avalanche			12
93	India	Dudhpokri lake west	1992-1993	88.1122	27.5547				14
94	India	Gya Lake	2014/8/6	77.6136	33.6175	Buried ice melting	1.28	470	32
95	Pakistan	Phander East	1993-1996	73.1981	36.0289				14
96	Pakistan	Ishkoman lake East	2002-2004	73.9	36.61				14
97	Kyrgyzstan	Teztor-1	1988/6/1	74.4314	42.5364			10	33
98	Kyrgyzstan	Teztor-1	1953/6/22	74.4314	42.5364		1.2	400	33
99	Kyrgyzstan	Teztor-2	2012/7/31	74.4275	42.5364	High temperature and/or heavy precipitation	0.2	340	33
100	Kyrgyzstan	Teztor-3	2005/7	74.4283	42.5378			20	33
101	Kazakhstan	Tuyuksu glacier lake	1973/7/15	77.085	43.0628	High temperature and/or heavy precipitation	0.76		34
102	Kazakhstan	No.35 glacier lake	1980/7/23	76.6017	42.9225	Buried ice melting	0.22		34
103	Kyrgyzstan	Angy-Say	1974/6/14	77.1489	41.9839	Ice tunnel drainage			35,36
104	Kyrgyzstan	Angy-Say	1975/6/17	77.1489	41.9839	Ice tunnel drainage			35,36
105	Kyrgyzstan	Angy-Say	1980/7/14	77.1489	41.9839	Ice tunnel drainage			35,36
106	Kyrgyzstan	Angy-Say	1980/6/25	77.1489	41.9839	Ice tunnel drainage			35,36
107	Kyrgyzstan	Kashka-Suu glacial lake	2006/7-2006/8	76.9956	41.8583	Ice tunnel drainage	0.194		35,36
108	Kyrgyzstan	Suuk-Tor glacial lake	1985	76.8661	41.9681	Ice tunnel drainage			35,36
109	Kyrgyzstan	No.112 glacier lake	2000-2002	77.5317	41.9611				35
110	Kyrgyzstan	Karateke Glacial lake	2013	76.8206	41.9778	Ice tunnel drainage	0.123		36
111	Kyrgyzstan	Jeruy Glacial lake	2014	76.8142	41.9867	Ice tunnel drainage	0.182		36
112	Kazakhstan	Sovetov	1975/8/19	77.0358	43.0411				34
113	Kazakhstan	Sovetov	1977/8/3	77.0358	43.0411		0.2		34
114	Kazakhstan	No.151 glacier lake	1970/7/14	77.1469	43.015				34
115	Kazakhstan	No.151 glacier lake	1971/7/12	77.1469	43.015				34
116	Kazakhstan	No.182 glacier lake	1974/7/22	77.2572	43.1114				34
117	Kazakhstan	No.182 glacier lake	1974/8/2	77.2572	43.1114				34
118	Kazakhstan	No.182 glacier lake	1979/6/21	77.2572	43.1114	Ice tunnel drainage	0.08		37
119	Kazakhstan	Tushinskiy	1982/9/2	79.9322	45.0792	Ice avalanche	2.7	2400	38
120	Kyrgyzstan	Zharsai	1977/7/3	77.4075	43.1522				34
121	Tajikistan	Dasht	2002/8/7	71.7286	37.2197		0.32		39
122	Tucumanistan	Archa-Bashi Glacier Lake	1998/7/7	71.9136	39.845		0.05		33
123	Kazakhstan	Tuyuksu glacier lake	1956/8/7	77.085	43.0628		0.035	32	40
124	Kazakhstan	Tuyuksu glacier lake	1951/8/20	77.085	43.0628		0.02		40
125	Kazakhstan	No.35 glacier lake	1986/7/5	76.6017	42.9225	Ice tunnel drainage	0.09	100	40
126	Kazakhstan	No.35 glacier lake	1988/6/24	76.6017	42.9225	Ice tunnel drainage	0.07	25	40

127	Kazakhstan	No.182 glacier lake	1973/7/15	77.2572	43.1114				40
128	Kazakhstan	No.182 glacier lake	1973/8/19	77.2572	43.1114				40
129	Kazakhstan	Zharsai	1977/6/28	77.4075	43.1522		0.46	7	40
130	Kazakhstan	Zharsai	1963/7/7	77.4075	43.1522		0.46	50	40
131	Kazakhstan	Zharsai	1958/7/6	77.4075	43.1522		0.2	20	40
132	Kazakhstan	Kargalinsky glacial lake	2015/7/23	76.9469	43.0208	Ice tunnel drainage			40
133	Kazakhstan	Kargalinsky glacial lake	2019/8/14	76.9469	43.0208	Ice tunnel drainage			40
134	Kazakhstan	N/N glacial lake	1993/7/6	77.2761	43.1458	Ice tunnel drainage			40
135	Kazakhstan	N/N glacial lake	2014/7/17	77.2761	43.1458	Ice tunnel drainage			40
136	Kazakhstan	Sportivny glacial lake	1979/6/21	77.3436	43.1672	Ice tunnel drainage	0.082	15	40
137	India	Rumbak	2021/8/22	77.4223	33.9997				41
138	Pakistan	Unnamed	2021/6/8	73.6379	36.3105				41
139	Kyrgyzstan	Toguz-Bulak	2019/8/8	77.2669	41.9776				42
140	Afghanistan	Pishkandah	2018/7/11	69.6245	35.4711				41
141	India	Unknown	2017/8/4	76.733	34.588				41
142	Afghanistan	Gandukh	2013/9/9	71.1289	36.4747				41
143	India	Chorog	2010/10/8	78.484	33.033				41
144	Kyrgyzstan	Western Zyndan	2008/7/24	77.0299	41.9478				34
145	Kyrgyzstan	Bogatyr	1985/7/23	77.3256	43.0207				40

Supplementary Table 3 | Reconstruction of drainage volume for the historical GLOFs after 1980. Glacial lake area change before and after GLOF was estimated based on the clear Landsat images. Glacial lake volume was respectively calculated based on different lake types, using the empirical equations fitted by Zhang et al.[43]. Focusing on the GLOFs drained after 1980, 65 events were successfully estimated. However, 28 events failed due to the no available remote sensing images or extremely small area change before and/or after drainage. Note, 19 values of drainage volume marked with an asterisk represent that they have reported real value in Supplementary Table 2. They were used to verify the reliability of those reconstructed values.

ID	Lake name	Lake type	Date of last clear image before outburst	Date of next clear image before outburst	Area before (km^2)	Area after (km^2)	Volume before (km^2)	Volume after (km^2)	Drainage volume (10^6m^3)	References
6	Cirenmaco	pro	1976-10-27		0.031					1
7	Cirenmaco	pro								4,5
16	Zarico	pro								1
17	Yindapuco	pro	1977-01-03	1987-12-22	1.047	0.645	47.642	23.057	24.58*	1,4
18	Ganxico	pro	1988-06-05	1988-10-27	0.435	0.173	12.754	3.189	9.56*	2,7
19	Xiaga	peri	1995-04-11	1995-09-02	0.159	0.136	2.621	2.029	0.59	8
20	Zanaco	peri	1995-05-25	1995-11-01	0.087	0	0.992	0	0.99	4
21	Chongbaxiaco	peri	2001-07-23	2003-11-18	0.773	0.452	32.267	13.744	18.52	4
22	Jialongco	peri	2001-02-02	2002-07-07	0.252	0.195	5.411	3.617	1.79	4,9
23	Jialongco	peri								4,9
24	Degaco	peri	2001-09-02	2003-11-18	0.14	0.106	2.122	1.372	0.75	4
25	Langco	extra	2007-05-14	2008-05-16	0.088	0.064	1.046	0.72	0.33	10
26	Zhemaico	peri	2009-05-19	2009-10-10	0.031	0.025	0.199	0.137	0.06	3
27	Coga	pro	2008-10-16	2009-09-17	0.491	0.173	15.319	3.2	12.12	4
28	Geiqu	peri	2010-06-21	2010-12-14	0.078	0	0.833	0	0.83	4
29	Gongbatongshaco	peri	2015-10-07	2016-07-14	0.019	0	0.091	0	0.09*	11
30	Zangla Tsho	peri	1994-07-30	1994-09-09	0.225	0.013	4.543	0.047	4.50	12
31	Langbu Tsho	peri	1992-07-04	1992-09-22	0.119	0.01	1.649	0.034	1.61	12
32	Ranzeranco	peri	2013-06-16	2013-09-12	0.592	0.247	21.114	5.245	15.87*	13
33	Gebumaco	pro	1992-09-04	1993-10-09	0.207	0.076	4.197	0.94	3.26	2
34	Dalonggongba	pro	2007-07-12	2008-06-28	0.202	0.156	4.028	2.727	1.30	2
35	Rejieco	peri	1992-04-16	1992-11-10	0.268	0.202	5.973	3.825	2.15	2
36	Zhalonggabu	pro	1995-11-01	1996-10-18	0.138	0.071	2.28	0.845	1.43*	2,14
37	Oubuguoco Upper	peri	2003-11-04	2004-10-21	0.09	0.051	1.061	0.426	0.63	2
38	Nalongzangbu Tributary	peri	2013-08-04	2014-08-23	0.117	0	1.594	0	1.59	2
39	Tulaco	peri	2014-10-17	2015-10-20	0.011	0	0.035	0	0.04	2
45	Sanga North	peri	1987-05-16	1987-11-08	0.185	0.089	3.332	1.043	2.29	14
46	Unnamed 2nd	peri	1997-09-07	1997-10-18	0.057	0.02	0.505	0.099	0.41	14
47	Dareco Sourth	pro	2001-02-02	2003-12-25	0.145	0.072	2.443	0.857	1.59	14
48	Jitang Sourth	pro	2002-06-10	2003-10-19	0.19	0.134	3.671	2.186	1.49	14
49	Yindapuco upper	peri	2015-10-25	2015-11-10	0.075	0.043	0.787	0.331	0.46	14
50	Zanglaco East	peri	2015-09-05	2016-05-16	0.297	0.213	7.03	4.161	2.87	14
51	Sangwangco East	peri	2007-07-08	2008-12-17	1.036	0.812	51.39	34.872	16.52	14
52	Baitang Weat	peri	1990-11-16	1991-10-09	0.063	0	0.605	0	0.60	14

54	Zanglaco West	peri	1994-12-30	1995-10-30	0.236	0	4.874	0	4.87	17
55	Jinwuco	pro	2020-03-27	2020-07-26	0.561	0.264	18.686	6.032	12.65*	18
56	Pasu Lake	peri								19
57	Pasu Lake	peri								19
58	Unknow2	peri	2018-06-18	2018-10-15	0.203	0.116	3.852	1.574	2.28	This study
59	Unknow1	peri	2017-06-06	2017-10-20	0.024	0	0.132	0	0.13	This study
60	Hezhen South	extra	2008-07-07	2009-10-14	0.193	0.121	2.58	1.516	1.06	14
67	upper Gangri Tsho1	extra	1997-11-01	1998-09-10	0.074	0.032	0.864	0.324	0.54	15
68	upper Gangri Tsho2	peri	1997-11-01	1998-09-10	0.485	0.426	15.389	12.5	2.89	17
71	Upper Luggye	supra	1991-05-09	1991-09-30	0.101	0.053	0.908	0.39	0.52	14
72	Upper Luggye	supra	2009-10-01	2010-10-04	0.133	0.084	1.304	0.712	0.59	14
73	Unamed 3rd	peri	1996-07-25	1999-09-20	0.043	0	0.323	0	0.32	14
74	Luggye Tsho	pro	1994-09-22	1994-11-09	1.219	0.929	59.851	39.813	20.04*	20
75	Lemthang Tsho	peri	2015-03-08	2015-10-18	0.064	0	0.611	0	0.61*	20
77	Nagma Pokhari	peri								22
78	Dig Tsho	peri	1979-01-06	1987-12-06	0.783	0.582	32.933	20.55	12.38*	23
79	Dig Tsho	peri	2015-04-23	2015-05-25	0.198	0.103	3.688	1.317	2.37	22
80	Chubung	peri	1990-04-18	1992-03-06	0.075	0	0.795	0	0.79	24
82	Tam Pokhari	peri	1998-02-19	1998-10-17	0.584	0.216	20.668	4.236	16.43*	20,26
83	Langmale lake	peri	2017-04-12	2017-11-06	0.134	0.035	1.982	0.233	1.75*	22
84	Zanglha North	peri	1998-06-11	1999-11-21	0.088	0.051	1.011	0.424	0.59	14
85	Lhotse Glaicer lake	supra	2015-05-25	2015-06-10	0.029	0	0.176	0	0.18	27
86	Lhotse Glaicer lake	supra	2016-05-27	2016-10-18	0.023	0	0.127	0	0.13	27
89	Unamed 4th	peri	2010-06-09	2012-12-13	0.006	0	0.016	0	0.02	17
90	Changri Shar Glacier lake	supra	2017-04-28	2017-10-21	0.233	0.144	2.71	1.446	1.26	29
91	Choradari	extra	2013-05-29	2013-06-30	0.037	0	0.392	0	0.39*	30,31
92	Kongyangmi La Tsho	peri	1994-10-31	1996-05-13	0.5	0.252	16.152	5.439	10.71	12
93	Dudhpokri lake west	pro	1991-11-08	1992-05-18	0.09	0.059	1.201	0.633	0.57	14
94	Gya Lake	pro	2014-07-10	2014-08-11	0.105	0.096	1.507	1.314	0.19*	32
95	Phander East	peri	1994-07-08	1996-07-29	0.03	0	0.184	0	0.18	14
96	Ishkoman lake East	pro	2001-07-11	2008-06-28	0.163	0	2.927	0	2.93	14
97	Teztor-1	peri								33
99	Teztor-2	peri	2010-08-28	2013-07-27	0.042	0	0.316	0	0.2*	33
100	Teztor-2	peri								33
102	No.35 glacier lake	peri								34
105	Angy-Say	peri								35,36
106	Angy-Say	peri								35,36
107	Kashka-Suu glacial lake	peri	2005-06-21	2007-09-07	0.061	0.031	0.576	0.194	0.38*	35,36
108	Suuk-Tor glacial lake	peri								35,36
109	No.112 glacier lake	peri	2001-06-26	2002-07-24	0.072	0.038	0.739	0.266	0.47	35
110	Karateke Glacial lake	peri	2011-09-26	2013-08-06	0.02	0	0.094	0	0.09*	36
111	Jeruy Glacial lake	peri	2013-10-09	2015-07-11	0.017	0	0.071	0	0.07*	36
119	Tushinskiy	peri								38
121	Dasht	peri	2002-08-05	2002-08-30	0.049	0	0.407	0	0.32*	39

122	Archa-Bashi Glacier Lake	peri	1997-10-18	1998-09-03	0.021	0	0.106	0	0.05*	33
125	No.35 glacier lake	peri								40
126	No.35 glacier lake	peri								40
133	Kargalinsky glacial lake	peri								40
134	N/N glacial lake	peri								40
135	N/N glacial lake	peri								40
137	Rumbak	peri	2018-08-28		0.005					41
138	Unnamed	peri								41
139	Toguz-Bulak	peri	2018-09-03		0.029					42
140	Pishkandah	peri	2018-07-02		0.011					41
141	Unknown	peri								41
142	Gandukh	pro	2012-10-03		0.16	0.07				41
143	Chorog	peri	2010-09-27		0.044					41
144	Western Zyndan	peri	2007-10-28		0.04					34
145	Bogatyr	peri	1978-08-16		0.426					40

Supplementary Table 4 | Relationship between GLOF's drainage volume and peak discharge. All these values are reported through disaster investigations or model estimates, except for two values of drainage volume obtained from Supplementary Table 3.

ID	Glacial lake	Lake type	Longitude (°)	Latitude (°)	Drainage volume ($10^6 m^3$)	Peak discharge ($m^3 \cdot s^{-1}$)	References
2	Qiongbihema Tsho	peri	88.9211	27.8469	12.4	1036	1
6	Cirenmaco	peri	86.0656	28.0669	18.9	16000	1
8	Longda Tsho	pro	85.3492	28.6169	10.8	3100	1
10	Damenlahaico	peri	93.0411	29.8675	3.7	2812	1,6
18	Ganxico	pro	96.5022	29.4631	5.4	1270	2,7
19	Xiaga	peri	91.9361	28.8022	0.59	38.3	8
29	Gongbatongshaco	peri	86.0636	28.0778	1.4	618	11
55	Jinwuco	pro	93.6317	30.3564	10	5602	18
75	Lemthang Tsho	peri	89.5806	28.0686	0.37	1562	20
76	Nare Lake	peri	86.8747	27.8369	4.9	1100	20,21
78	Dig Tsho	peri	86.5856	27.8744	5	1600	23
81	Chukhung	peri	86.8875	27.8886	5	1915	25
82	Tam Pokhari	peri	86.8444	27.7414	18	10000	20,26
83	Langmale lake	peri	87.1381	27.8111	1.3	4400	22
86	Lhotse Glaicer lake	supra	86.8983	27.9058	0.13	210	27
91	Choradari	extra	79.0608	30.7472	0.43	1352	30,31
94	Gya Lake	pro	77.6136	33.6175	1.28	470	32
99	Teztor-2	peri	74.4275	42.5364	0.2	340	33
119	Tushinskiy	peri	79.9322	45.0792	2.7	2400	38

Supplementary Table 5 | Inundation area, potential disaster intensity and exposure of GLOFs in the Third Pole aggregated into the GTN-G regions. The exposure was quantified by building, hydropower, farmland, road, and bridge.

GTN-G regions	Inundation area (km^2)	Reginal potential disaster intensity	Number of glacial lakes with high outburst potential	Building	Hydropower	Farmland (km^2)	Road (km)	Bridge
Altai	74.1	0.042	21	69	0	0	29	10
Hissar Alay	40.3	0.479	15	429	0	1.4	66	100
Pamir	290.3	0.16	62	1430	0	8.1	255	120
West Tian	510.5	0.211	120	10144	3	10.9	554	308
East Tian	360.6	0.155	87	3048	5	24.1	393	264
West Kunlun	110.6	0.022	7	0	0	0	1	0
East Kunlun	250.7	0.009	23	13	0	0	40	3
Qilian Shan	216.9	0.018	25	20	0	0	53	10
Inner Tibet	1513.4	0.08	164	1612	1	18.1	489	216
SE Tibet	470.7	0.383	123	10042	1	28.1	775	621
Hindu Kush	125.8	0.516	38	2858	7	16.7	170	329
Karakoram	210.2	0.193	64	1214	0	5.9	90	107
Western Himalaya	638.2	0.209	162	4555	19	17.4	424	425
Central Himalaya	374	0.203	126	3659	28	6	349	282
Eastern Himalaya	850.1	0.253	355	12671	40	49.3	942	914
Henduan Shan	316.4	0.237	107	4044	1	8	375	329

Supplementary Table 6 | Inundation area, potential disaster intensity and exposure of GLOFs in the Third Pole aggregated into the national areas. The exposure was quantified by building, hydropower, farmland, road, and bridge.

Countries	Inundation area (km^2)	Reginal potential disaster intensity	Number of glacial lakes with high outburst potential	Building	Hydropower	Farmland (km^2)	Road (km)	Bridge
China	4080.3	0.191	880	25836	13	124.3	2940	2127
Bhutan	349.8	0.149	70	2738	3	5.1	211	90
Nepal	422.4	0.231	121	5840	43	7.9	303	385
India	422.4	0.239	164	4039	33	12	427	366
Pakistan	199.2	0.489	60	4274	8	20	187	407
Afghanistan	131	0.405	31	1514	2	7.6	167	165
Tajikistan	201.2	0.227	51	1417	0	7.3	210	158
Kyrgyzstan	387.9	0.239	74	955	0	7	293	172
Kazakhstan	101.2	0.207	32	9126	3	2.8	252	158
Mongolia	40.6	0.043	10	47	0	0	15	9
Russia	17.8	0.067	6	22	0	0	12	1

**Supplementary Table 7 | ** Gullies with the maximum GLOF probability greater than 0.4. Several necessary factors were counted according to the sub-basins where they were located.

ID	Basin name	Region	Central latitude (°)	Central longitude (°)	Number of glacial lakes with high outburst potential	Transboundary	Maximum GLOF probability
1	Tomor	Eastern Himalaya	27.6421	87.8272	14	No	0.33
2	Gaqu	Southeastern Tibet	31.6872	95.1008	8	No	0.57
3	Meiqu	Southeastern Tibet	30.9615	94.8777	6	No	0.38
4	Nimuqu	Southeastern Tibet	31.1301	93.9798	10	No	0.29
5	Remaqu	Southeastern Tibet	31.6651	94.4464	6	No	0.29
6	Teesta	Eastern Himalaya	27.0849	88.6477	46	No	1.00
7	Mangdechhu	Eastern Himalaya	27.5059	90.6489	13	No	0.33
8	Puna Tsang Chhu	Eastern Himalaya	27.9116	89.8689	25	No	0.52
9	Nyamjang Chhu	Eastern Himalaya	28.0254	91.6922	17	Yes	0.48
10	Luozhaxiongqu	Eastern Himalaya	28.2769	90.7402	29	Yes	0.71
11	Dudh Kosi	Eastern Himalaya	27.6323	86.7265	31	No	0.57
12	Aru	Eastern Himalaya	28.1105	87.1684	49	Yes	0.71
13	Pengqu	Eastern Himalaya	27.8677	86.2431	26	Yes	0.62
14	Boiqu	Eastern Himalaya	28.1216	85.9387	18	Yes	0.71
15	Yeruzangbu	Eastern Himalaya	28.36	88.1954	23	No	0.43
16	Gyirong	Central Himalaya	28.2623	85.3022	28	Yes	0.71
17	Duoqu	Eastern Himalaya	28.102	92.4381	14	No	0.33
18	Gongrigabuqu	Henduan Shan	29.0158	96.635	7	No	0.38
19	Jingzhuqu	Henduan Shan	29.5929	95.5219	6	No	0.48
20	Yigongzangbu	Southeastern Tibet	30.202	94.676	12	No	0.38
21	Zepuqu	Henduan Shan	29.9235	95.9756	55	No	0.67
22	Xiongqu	Southeastern Tibet	30.6717	93.6972	31	No	0.67
23	Zhulaqu	Southeastern Tibet	30.1327	93.7453	23	No	0.38
24	Majiazangbu	Central Himalaya	30.2845	81.3154	24	Yes	0.52
25	Nianchu	Eastern Himalaya	28.1989	89.346	20	No	0.24
26	Tesike	West Tianshan	42.7985	80.4558	17	Yes	0.48
27	Jingou	East Tianshan	43.9512	85.3614	7	No	0.48
28	Peiku	Central Himalaya	28.7785	85.6724	11	No	0.52

Supplementary References

1. Xu, D. et al. Studies on catastrophes of glacial debris flow and glacial lake outburst flood in China (in Chinese). *Journal of Glaciology and Geocryology* **10**, 284–289 (1988).
2. Liu, J. et al. An overview of glacial lake outburst flood in Tibet, China (in Chinese). *Journal of Glaciology and Geocryology* **41**, 1335–1347 (2019).
3. Liu, J. et al. Characteristics of glacier-lake breaks in Tibet (in Chinese). *Journal of Catastrophology* **23**, 55–60 (2008).
4. Yao, X. et al. Study on the glacial lake outburst flood events in Tibet since the 20th century (in Chinese). *Journal of Natural Resources* **29**, 1377–1390 (2014).
5. Chen, N. et al. On the water hazards in the trans-boundary Kosi River basin. *Nat. Hazards Earth Sys. Sci.* **13**, 795–808 (2013).
6. Lv, R. et al. Debris flow induced by ice lake burst in the Tangbulang gully, Gongbujiangda, Xizang (Tibet) (in Chinese). *Journal of Glaciology and Geocryology* **8**, 61–67 (1986).
7. Li, D. et al. Bursting of the Midui moraine lake in Bomi, Xizang (in Chinese). *Mountain Research* **10**, 219–224 (1992).
8. Li, D. et al. Preliminary study on the cause of glacier floods on May 26, 27, 28 in Yalong River (in Chinese). *Tibet's Science and Technology* 37–39 (1995).
9. Chen, X. et al. Debris flows of Chongdui gully in Nyalam county, 2002: Cause and control (in Chinese). *Journal of Glaciology and Geocryology* **2**, 776–781 (2006).
10. Mo, Y. et al. The causes and prevention countermeasures of 2007-08-10 large-scale debris flow hazard in Cuona County, Tibet (in Chinese). *Journal of Institute of Disaster-Prevention Science and Technology* **10**, 43–46 (2008).
11. Chen, N.et al. Small outbursts into big disasters: Earthquakes exacerbate climate-driven cascade processes of the glacial lakes failure in the Himalayas. *Geomorphology* **422**, 108539 (2023).
12. Nie, Y. et al. An inventory of historical glacial lake outburst floods in the Himalayas based on remote sensing observations and geomorphological analysis. *Geomorphology* **308**, 91–106 (2018).
13. Sun, M. et al. The cause and potential hazard of glacial lake outburst flood occurred on July 5, 2013 in Jiali County, Tibet (in Chinese). *Journal of Glaciology and Geocryology* **36**, 158–165 (2014).
14. Veh, G. et al. Unchanged frequency of moraine-dammed glacial lake outburst floods in the Himalaya. *Nat. Clim. Chang.* **9**, 379–383 (2019).
15. Komori, J. et al. Glacial lake outburst events in the Bhutan Himalayas. *Global Environ. Res.* **16**, 59–70 (2012).
16. Tong L. et al. Characteristics of the Dacanggou debris flow induced by breakout of glacier-lake in the Pengqu basin Dirin County of Tibet (in Chinese). *The Chinese Journal of Geological Hazard and Control* **30**, 34–48 (2019).
17. Zhang, T. et al. Glacial lake outburst floods on the High Mountain Asia: a review (in Chinese). *Journal of Glaciology and Geocryology* **43**, 1673–1692 (2021).
18. Zheng, G.et al. The 2020 glacial lake outburst flood at Jinwuco, Tibet: causes, impacts, and implications for hazard and risk assessment. *Cryosphere* **15**, 3159–3180 (2021).
19. Zhu, Y. et al. Glacier geo-hazards along China-Pakistan International Karakoram Highway (in Chinese). *Journal of Highway and Transportation Research and Development* **31**, 51–59 (2014).
20. Gurung, D. et al. Lemthang Tsho glacial Lake outburst flood (GLOF) in Bhutan: cause and impact. *Geoenvironmental Disasters* **4**, (2017).
21. Buchroithner, M. et al. Monitoring of Recent Geological Events in the Khumbu Area (Himalaya, Nepal) by Digital Processing of Landsat MSS Data. *Rock Mechanics* **15**, 181–197 (1982).
22. Byers, A. et al. A rockfall-induced glacial lake outburst flood, Upper Barun Valley, Nepal. Landslides **16**, 533–549 (2018).
23. Vuichard, D. et al. The 1985 catastrophic drainage of a moraine-dammed Lake, Khumbu Himal, Nepal: Cause and consequences. *Mt. Res. Dev.* **7**, 91–110 (1987).
24. Bajracharya, S.et al. Glaciers, glacial lakes and glacial lake outburst floods in the Mount Everest region, Nepal. *Ann. Glaciol.* **50**, 81–86 (2009).

25. Westoby, M. et al. Modelling outburst floods from moraine-dammed glacial lakes. *Earth Sci. Rev.* **134**, 137–159 (2014).
26. Osti, R. et al. Hydrodynamic characteristics of the tam Pokhari glacial Lake outburst flood in the Mt. Everest region, Nepal. *Hydrol. Process.* **23**, 2943–2955 (2009).
27. Rounce, D. et al. Brief communication: Observations of a glacier outburst flood from Lhotse Glacier, Everest area, Nepal. *Cryosphere* **11**, 443–449 (2017).
28. Byers, A. et al. Reconstructing the History of Glacial Lake Outburst Floods (GLOF) in the Kanchenjunga Conservation Area, East Nepal: An Interdisciplinary Approach. *Sustainability* **12**, (2020).
29. Miles, E. et al. Glacial and geomorphic effects of a supraglacial lake drainage and outburst event, Everest region, Nepal Himalaya. Cryosphere **12**, 3891–3905 (2018).
30. Das, S. et al. Glacial lake outburst flood at Kedarnath, Indian Himalaya: a study using digital elevation models and satellite images. *Nat. Hazard* **77**, 769–786 (2015).
31. Allen, S. et al. Lake outburst and debris flow disaster at Kedarnath, June 2013: hydrometeorological triggering and topographic predisposition. *Landslides* **13**, 1479–1491 (2015).
32. Majeed, U. et al. Recession of Gya Glacier and the 2014 glacial lake outburst flood in the Trans-Himalayan region of Ladakh, India. *Sci. Total Environ.* **756**, 144008 (2021).
33. Erokhin, S. et al. Debris flows triggered from non-stationary glacier lake outbursts: the case of the Teztor Lake complex (Northern Tian Shan, Kyrgyzstan). *Landslides* **15**, 83–98 (2017).
34. Narama, C. et al. Current state of glacier changes, glacial lakes, and outburst floods in the Ile Ala-Tau and Kungöy Ala-Too ranges, northern Tien Shan Mountains. *Annals of Hokkaido Geography* **84**, 22–32 (2009).
35. Narama, C. et al. The 24 July 2008 outburst flood at the western Zyndan glacier lake and recent regional changes in glacier lakes of the Teskey Ala-Too Range, Tien Shan, Kyrgyzstan. *Nat. Hazard Earth Sys. Sci.* **10,** 647–659 (2010).
36. Narama, C. et al. Large drainages from short-lived glacial lakes in the Teskey Range, Tien Shan Mountains, Central Asia. *Nat. Hazard Earth Sys. Sci.* **18**, 983–995 (2018).
37. Bolch, T. et al. Identification of potentially dangerous glacial lakes in the northern Tien Shan. *Nat. Hazard* **59**, 1691–1714 (2011).
38. Petrakov, D. et al. Debris flow hazard of glacial lakes in the Central Caucasus. In: Chen, Y., Major, S. (Eds.), Debris-Flow Hazards Mitigation: Mechanics, Prediction, and Assessment. Millpress, Netherlands, pp. 703–714 (2007).
39. Mergili, M. et al. Regional-scale analysis of lake outburst hazards in the southwestern Pamir, Tajikistan, based on remote sensing and GIS. *Nat. Hazard Earth Sys. Sci.* **11**, 1447–1462 (2011).
40. Medeu, A. et al. Moraine-dammed glacial lakes and threat of glacial debris flows in South-East Kazakhstan. *Earth Sci. Rev.* **229**, (2022).
41. Shrestha, F. et al. A comprehensive and version controlled database of glacial lake outburst floods in High Mountain Asia. *Earth Sys. Sci. Data* **15**, 3941–3961 (2023).
42. Daiyrov, M. et al. Formation and outburst of the Toguz-Bulak glacial lake in the northern Teskey Range, Tien Shan, Kyrgyzstan. *Geosciences* **10**, 468 (2020).
43. Zhang, T. et al., A conceptual model for glacial lake bathymetric distribution. *Cryosphere* (2023) (in Discussion).
